# Dysbacteriosis of the Intestinal Flora Is an Important Reason for the Death of Adult House Flies Caused by *Beauveria bassiana*

**DOI:** 10.3389/fimmu.2020.589338

**Published:** 2021-01-26

**Authors:** Ruiling Zhang, Shuo Feng, Xiaochen Xie, Zhendong Huang, Qing Wan, Shumin Wang, Zhong Zhang

**Affiliations:** ^1^Collaborative Innovation Center for the Origin and Control of Emerging Infectious Diseases, Shandong First Medical University and Shandong Academy of Medical Sciences, Taian, China; ^2^School of Basic Medical Science, Shandong First Medical University and Shandong Academy of Medical Sciences, Taian, China

**Keywords:** *Beauveria bassiana*, *Musca domestica*, intestinal flora, dysbacteriosis, pathogenic fungi

## Abstract

*Beauveria bassiana* is one of the most widespread insect pathogens and can be used in the biological control of agricultural, forestry and medical pests. The mechanisms by which *B. bassiana* leads to mortality in different host insects are also different. For house flies, *B. bassiana* has strong virulence, but its microecological mechanism is not clear. In this paper, the virulence of three strains of *B. bassiana* (TB, CB and BB) isolated from different hosts to house flies was studied. The results showed that the three strains of *B. bassiana* had strong pathogenicity to house fly adults. Specifically, TB was the strongest, CB was the second strongest, and BB was the weakest, with maximum lethal effects on house fly populations 5, 6, and 7 days after infection, respectively. Further study showed that the intestinal flora of house flies was disordered 3, 4, and 5 days after *B. bassiana* TB, CB and BB strain infection, respectively. Intestinal flora dysbacteriosis may be an important reason for the death of house flies caused by *B. bassiana*. After infection, the negative interaction ratio of bacteria in the house fly intestine decreased, and the stronger the virulence was, the lower the negative interaction ratio was. The time from *B. bassiana* infection to intestinal flora dysbacteriosis was not fixed. We named this period the “spring stage”. The stronger the virulence of the *B. bassiana* strain was, the shorter the “spring stage” was. Therefore, the “spring stage” can be used as a virulence marker for evaluating the pathogenicity of different *B. bassiana* strains.

## Introduction

The house fly, *Musca domestica* L. (Diptera: Muscidae), is an important medical insect that interferes with human life by causing annoyance, irritation, and food spoilage. It can also mechanically transport pathogenic disease in both people and animals. Over 130 pathogens (including fungi, bacteria, viruses and parasites) have been identified in house flies, causing intestinal infections, eye infection, certain skin infections, polio, bird flu and other illnesses (1–3). House flies have also been found to carry multidrug-resistant bacteria, including human pathogens, from hospital environments to nonhospital areas, creating serious problems for residents (4–6). In addition, the high population density of the house fly in poultry farms causes irritation and annoyance to employees (7). Therefore, house fly is an important pest that affect public health and the healthy development of the livestock breeding industry.

The misuse of insecticides often leads to environmental pollution and ill effects on public health, so house fly control has changed from traditional chemical control to integrated pest management (IPM). Biological control is a green and environmentally friendly pest control method that is also widely used in IPM (8). Biological control includes the use of botanical pesticides, fungal/bacterial pathogens and parasitoids/predators to control house fly larvae, pupae or adults (9, 10).

For house flies, *Beauveria bassiana* is one of the most common fungal parasites. The first reported natural occurrence of *B. bassiana* in the house fly was in 1990 (11). Since then, many studies have been conducted on the attempted use of *B. bassiana* against house flies in laboratory and field experiments (12–17).

*B. bassiana* is deployed for house fly control in bait and spray forms (16, 18) and can take up to 7 days, causing significant mortality (12). Some *B. bassiana* strains have been processed into commercial fungal formulations (16, 19).

*B. bassiana* infection of host insects can be divided into three steps: adhesion of the spore, penetration through the cuticle, and establishment within the host (20, 21). *B. bassiana* spores attached to the epicuticle of insects are accompanied by electrostatic and hydrophobic interactions. Subsequent to attachment, spores germinate on the insect cuticle, and then the germinated conidia (germ tube) penetrate into the cuticle. After penetration, fungi inhibit the host immune system and grow in the hemocoel, taking up nutrients, producing toxins, destroying host cells and eventually killing the insect (22–24). After infection, the gut bacteria are also altered by *B. bassiana*, such as in mosquitoes (25), cockroaches (5), *Delia antiqua* (26) and locusts (27). These studies were focused on one *B. bassiana* strain or different strains isolated from one host to a certain insect. However, no study has examined the relationship between the virulence of different *B. bassiana* strains and the composition of the intestinal bacteria of infected insects.

The efficacy of application of *B. bassiana* is dependent on the strain, dosage and formulation. Laboratory virulence evaluations typically involve forced-contact exposure methods, such as the immersion or dipping of the host insects into the spore formulations and even injection of the spores (14, 17, 21). Such methods are effective but unnatural and are likely to produce different results compared with those of field applications (16). Therefore, the bait method was used in this study, which is more natural than the forced-contact exposure methods. After *B. bassiana* exposure using the bait method, the intestinal bacteria of house flies were detected through the 16S rRNA high-throughput sequencing method. Therefore, the purpose of this paper is to establish the relationship between the virulence of *B. bassiana* and its ability to regulate the composition of host intestinal bacteria and to explore the microbiological mechanism by which *B. bassiana* kills host insects.

## Methods

### House Fly Rearing

The house fly strain was reared in the vector biological laboratory of Shandong First Medical University for approximately 15 years without exposure to pesticides or entomopathogenic fungi. Adult *M. domestica* were reared in gauze cages (30 cm × 30 cm × 30 cm) with mesh screens on opposite sides and a cloth sleeve opening at the front. The adult flies were provided with brown sugar in Petri dishes as a diet and allowed water *ad libitum*. After 2 to 3 days of feeding, clear plastic cups containing the larval diet were placed in the cages as an egg laying substrate. The diet was composited with sterilized wheat bran, dry milk powder and sterilized water (45.5:4.5:40), mixed to paste in proportion. When eggs became visible on the sides of cups or attached to the food, the cups were removed and kept separated for larval development. The larval food was changed every day. The insects were kept in an artificial climate chest at 28 ± 1°C, a 45% to 55% relative humidity (RH) and a photoperiod of LD 16:8 h.

### Sources of *Beauveria bassiana*

Three strains of *B. bassiana* were collected from naturally infected insects. *B. bassiana* BB was isolated from naturally infected cicada *Cryptotympana atrata* Bsg in Bashangou of Mount Tai, Shandong Province (117.10°E, 36.27°N); *B. bassiana* CB was isolated from naturally infected stink bug *Cyclopelta parva* Sddy on the campus of Shandong First Medical University, Taian city, Shandong Province (117.09°E, 36.13°N); and *B. bassiana* TB was isolated from naturally infected longicorn larvae of *Anoplophora glabripennis* Pzs at Puzhao Temple, Taian city, Shandong Province (117.12°E, 36.21°N) (**Figure S5**).

### *B. bassiana* Infection and Virulence

Fungi were cultured on PDA at 27°C for 7 days before use in experiments. The fungal conidia were collected from the mycelial surface in different plates, diluted with sterile water containing 0.05% Tween 80 to approximately 1×10^10^ conidia/ml and stored at 4°C. Approximately 10 μl conidial suspensions were placed with a micropipette into a 48-well plate containing 1% low-melting-point agar gel. The plate was placed at 27°C for 12 h, and the germination rate of spores was examined using a microscope. The spores with a germination rate higher than 90% were used for subsequent experiments. The spores from different PDA plates were examined in 3 wells as repeats. The qualified fungal suspensions were vortex mixed vigorously before the bioassay.

The activity of the three strains of *B. bassiana* (TB, CB, BB) against *M. domestica* adults was determined independently using the bait method (14). The 40-ml spore suspension (prepared by sterile Tween 80 solution to a concentration of approximately 10^9^ spores/ml) was mixed with 50 g wheat bran (containing 4.5 g powdered milk) as bioassay baits for house flies. For each group, the baits were put into gauze cages (30 cm × 30 cm × 30 cm) containing approximately 150 of 12 h-emerged house flies. Sterile water was provided as drinking water. The baits and water were changed every day. The experiment was carried out in three cages independently, and sampled in each cage every day, respectively, as replications. The house flies fed baits without *B. bassiana* spores were used as controls. The dead house flies in different cages were identified and collected daily until 9 days after treatment. The collected dead house flies were maintained at over 90% RH in an incubator at 28 ± 1°C. Dead house flies with visible fungal growth on their body surface were considered to have died of fungal infection.

Furthermore, in order to analyze the amount of feces excreted by male and female house flies, and the amount of eggs oviposited by female house flies fed on different baits, the 40-ml spore suspension (prepared by sterile Tween 80 solution to a concentration of approximately 109 spores/ml) was mixed with 48 g wheat bran (containing 4.5 g powdered milk) and 2 g red natural edible pigment (RNEP) (SUGARMAN^®^, China) as bioassay baits for house flies. For each group, the baits were put into gauze cages (30 cm × 30 cm × 30 cm) containing approximately 50 female and 50 male 12 h-emerged house flies. Sterile water containing 2% RNEP (SUGARMAN^®^, China) was provided as drinking water. The baits and water were changed every day. The experiment was carried out in three cages independently, and sampled in each cage every day, respectively, as replications. The house flies fed baits without *B. bassiana* spores were used as controls. Every day, 10 female and male house flies were randomly removed from each cage and placed in a Petri dish with clean filter paper on the bottom. They were allowed to defecate for 2 h, and the amount of red feces produced by house flies on the filter paper was assessed. After the test, the house fly was put back into the original cage. The eggs oviposited in the bait were counted daily when baits were changed.

### Sample Collection for Intestinal Bacterial Detection

**Figure 1:**
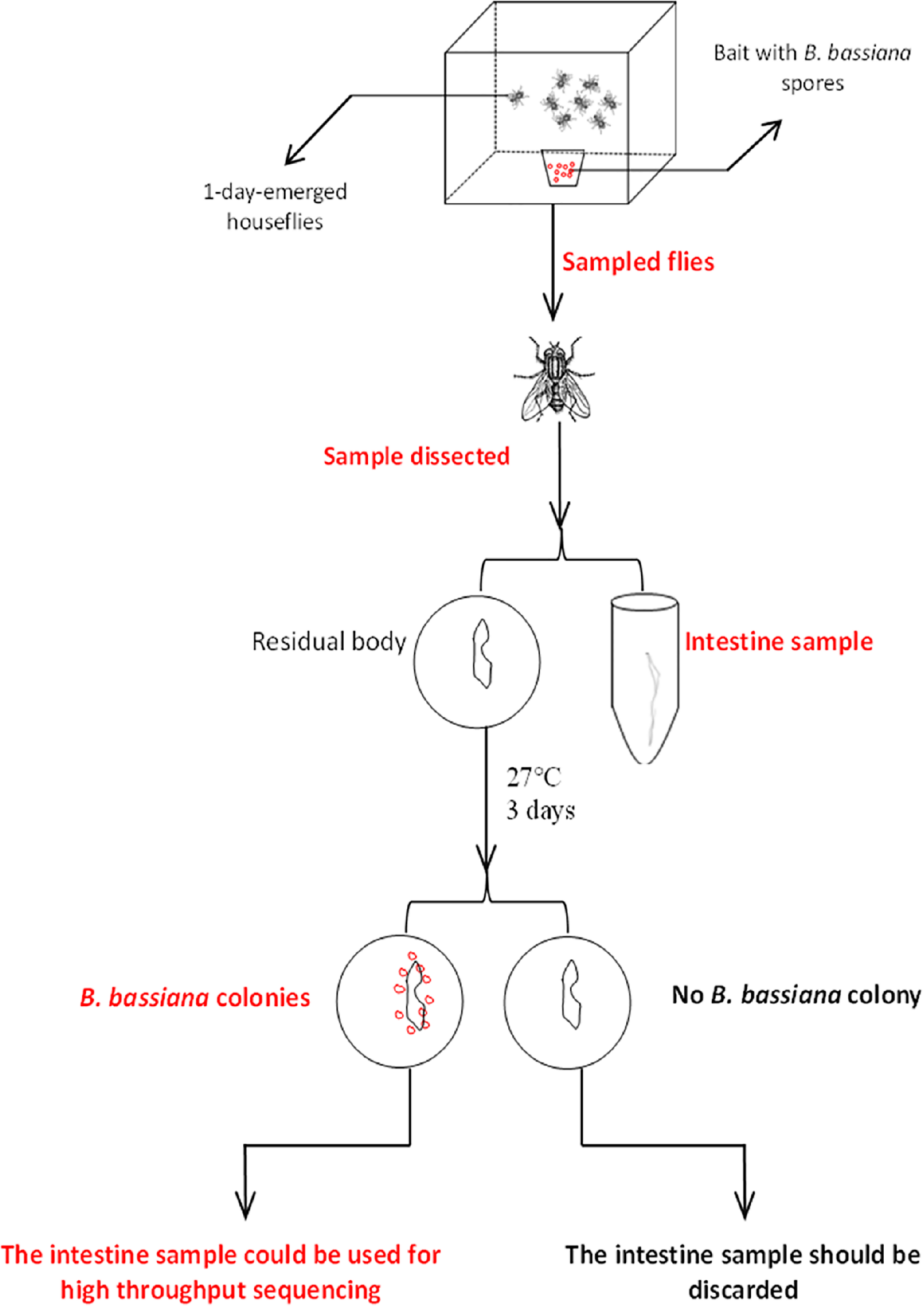
Sample screening process.

Twenty house flies from each cage were sampled for 5 days continuously. Each sampled house fly was starved for 4 h to remove food, and then the surface was thoroughly cleaned with sterile water. Then, the whole intestine was dissected under sterile conditions. Once the intestinal surface was broken, the sample could not be used for subsequent tests. The dissected intestine was washed with sterile water 3 times and then placed in sterile centrifuge tubes containing sterile normal saline, with one sample for each tube, and the sample number was marked. After removing the intestine, the residual body of each house fly was placed on PDA medium and then placed in an incubator at 28 ± 1°C for approximately three days. Then, we observed whether *B. bassiana* colonies grew around or on the residual body. If colonies appeared, the house fly was considered successfully parasitized, and the corresponding intestinal sample was used for 16S rRNA high-throughput sequencing to detect intestinal bacterial composition. If there was no *B. bassiana* colony growth, the sample was excluded, and the corresponding intestinal sample was not used for the subsequent experiment. The house flies that died within 2 h were used as candidate samples and verified using the above method. However, house flies that died after more than 2 h were not chosen as a sample. Five verified intestinal samples were mixed together as a sampling unit. Each sample unit was a pool containing five intestines for *B. bassiana* BB-, CB- and TB-infected house flies or controls. Three units as 3 repetitions were used for 16S rRNA high-throughput sequencing of intestinal bacteria (**Figure 1**).

### DNA Extraction

The intestinal samples were homogenized in a tissue lyser (Qiagen, Hilden, Germany) followed by genomic DNA isolation using the Wizard Genomic DNA Purification Kit (Promega; A1120) according to the manufacturer’s instructions, with DNA suspended in 30 μl nuclease-free water. The concentration and quality of extracted DNA were assessed using a NanoDrop 2000 spectrophotometer (Thermo Fisher Scientific, Waltham, MA, USA) and 2% agarose gel electrophoresis, respectively. Extracted DNA was stored at −20°C until further processing.

### Illumina Sequencing and Bioinformatics Analysis

The hypervariable V3-V4 region of the bacterial 16S rRNA gene was amplified with the primers 341F (5′-CCTAYGGGRBGCASCAG-3′) and 806R (5′-GGACTACNNGGGTATCTAAT-3′). Twenty-microliter PCR mixtures were set up with 4 μl 5 × FastPfu buffer, 2 μl deoxynucleoside triphosphates (dNTPs) (2.5 mM), 0.8 μl each primer, 0.4 μl FastPfu polymerase, and 10 ng template DNA. Reactions proceeded in a GeneAmp 9700 (ABI) thermocycler with 95°C for 5 min; 27 cycles of denaturation at 95°C for 30 s, annealing at 55°C for 30 s, and elongation at 72°C for 45 s, followed by additional elongation at 72°C for 10 min; and a dissociation stage at the end of the run.

PCR products were detected by 2% agarose gel electrophoresis and purified using the QIAquick Gel Extraction Kit (Qiagen). Library pools were constructed with equal amounts of each PCR product by using the TruSeq Nano DNA LT Sample Prep Kit (Illumina), which was amplified through paired-end sequencing on the Illumina MiSeq PE300 platform.

Quality control of the original data was carried out using Trimmomatic v0.39 software (http://www.usadellab.org/cms/index.php?page=trimmomatic). Based on the overlap (minimum: 10 bp) between PE reads after quality control, PE reads were assembled using FLASH v1.2.11 software (FLASH: fast length adjustment of short reads to improve genome assemblies). QIIME v1.9.1 software (QIIME allows analysis of high-throughput community sequencing data) was adopted for processing, and VSEARCH v2.14.1 software (VSEARCH: a versatile open source tool for metagenomics) was used for detecting chimeric sequences. Based on a sequence similarity level of 97%, the UCLUST method in QIIME software was employed to perform OTU clustering analysis. On the basis of the Silva reference database (Release 138), taxonomic annotations were made for the OTUs in each sample. The Shannon, Simpson, Chao1, and Ace indexes of microbial communities were calculated by mothur (https://mothur.org/). LEfSe software (https://bitbucket.org/nsegata/lefse/src/default/) was used to estimate the abundance differences among microbial species in samples.

Principal coordinate analysis (PCoA) based on Bray-Curtis dissimilarity and an unweighted pair group method with arithmetic mean (UPGMA) tree based on unweighted UniFrac phylogenetic distances were used to determine the difference in bacterial community beta diversity in different samples. Bray-Curtis ordination is an effective strategy for the analysis of multivariate ecological data. Fast UniFrac facilitates high-throughput phylogenetic analyses of microbial communities, including analysis of pyrosequencing and PhyloChip data.

### Statistical Analysis

Paired-end reads were assigned to samples based on their unique barcodes and truncated by cutting off the barcode and primer sequence. Then, the paired-end reads were merged into longer single sequences using FLASH (v1.2.11) (Magoč and Salzberg 2011). Quality filtering was performed on the raw tags under specific filtering conditions to obtain high-quality clean tags (28) according to the QIIME (v1.8.0) (29) quality-control process. OTUs were clustered with a 97% similarity cut-off using UPARSE (v7.0.1090) (30). Chimeric sequences were detected and removed using UCHIME (v4.2.40) (Edgar et al., 2011). Representative sequences from each OTU were screened for further annotation. For each representative sequence, the Greengenes database (31) was used with RDP Classifier (v2.2) (32) to obtain taxonomic information. Microbial diversity was analyzed using QIIME v1.8.0 and displayed using R software (v3.0.3) (29). The alpha diversity analysis included observed species, Ace and Chao1 estimators, and the Simpson and Shannon diversity indexes. The mean and standard deviation values of variables were calculated using Microsoft Excel 2016. Before the analysis of variance (ANOVA), the homogeneity of the variances was visually verified by plotting the residuals against the predicted values. ANOVA was performed to determine the effects of *B. bassiana* infection on the Ace, Chao1, Simpson and Shannon indexes using SAS software (SAS Institute, 1999). Fisher’s least significant difference approach was used to assess the mean differences among treatments and their interactions at *p*< 0.05.

## Results

### Pathogenicity of Three *B. bassiana* Strains to the House Fly

**Figure 2:**
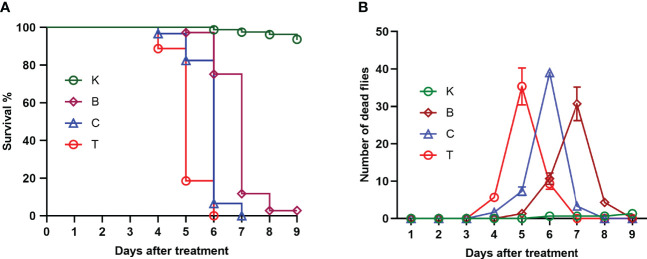
Survival curve **(A)** and number of deaths per day **(B)** of uninfected house flies and house flies infected by three *Beauveria bassiana* strains. K: Control; B, C and T: *Beauveria bassiana* strain BB-, CB- and TB-infected house flies.

**Figure 3:**
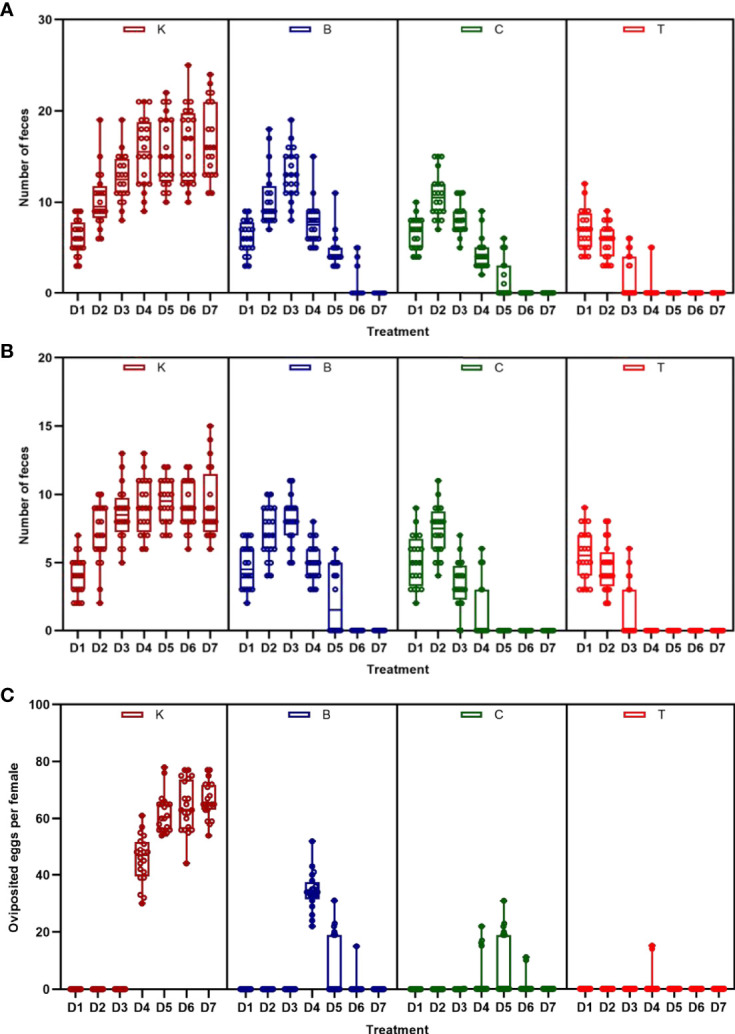
Amount of feces and eggs excreted by house flies after eating different baits. **(A)** Amount of feces excreted by female house flies after eating different *B. bassiana* strain baits and control baits blended with red natural edible pigment (SUGARMAN^®^, China). **(B)** Amount of feces excreted by male house flies after eating different *B. bassiana* strain baits and control baits blended with red natural edible pigment (SUGARMAN^®^, China). **(C)** Fecundity per female house fly fed on different baits; K: Control; B, C and T: *Beauveria bassiana* strain BB-, CB- and TB-infected house flies.

The three groups of house flies infected by *B. bassiana* strains BB, CB, and TB and the control house flies were referred to as groups B, C, T and K, respectively. The three *B. bassiana* strains had different virulence to house flies, and *B. bassiana* strain TB had the strongest virulence, followed by strains CB and BB. The peak death of house flies caused by TB, CB and BB occurred on the 5th, 6th, and 7th days after exposure, respectively. After treatment for 6 and 7 days, all the house flies in the T and C groups died, but the cumulative mortalities in the B and K groups were 97.25% and 6.29% until 9 days after treatment, respectively (**Figure 2**). Furthermore, feces and eggs excreted by adult house flies fed on different baits were analyzed. The amount of feces of *B. bassiana*-infected male and female adult house flies declined compared with the controls; group T excreted the smallest amount of feces, followed by groups C and B (**Figures 3A, B**). The number of eggs oviposited by infected house flies was significantly smaller than that in group K, and group T house flies laid almost no eggs (**Figure 3C**). 16S rRNA gene sequencing analysis and taxon generation of different house fly groups.

*Pantoea* in the intestine of adult house flies was the dominant taxon after infection by *B. bassiana* BB (group B) for 5 days, representing approximately 76.05 ± 1.89% of the total bacteria. *Cyanobacteria_c_norank* was the dominant taxon in the intestines of adult house flies after infection by *B. bassiana* CB (group C) for 4 days and *B. bassiana* TB (group T) for 3 days, accounting for approximately 93.65 ± 1.40% and 94.68 ± 2.12% of the total bacteria, respectively. Therefore, after infection with *B. bassiana* BB, CB and TB for 5, 4 and 3 days, the intestinal flora in the house flies was disturbed. The death peak of house flies infected by *B. bassiana* strains BB, CB and TB occurred 7, 6 and 5 days after infection, respectively. Therefore, we can conclude that the time interval between intestinal flora disorder induced by *B. bassiana* infection and the population death peak was 2 days, and the phenomenon of intestinal flora disorder occurred first. However, for different strains of *B. bassiana*, the time from exposure to intestinal flora disturbance was variable. We named the stage from *B. bassiana* infection to house fly intestinal flora disturbance the “spring stage”. The “spring stage” can be used as a marker of *B. bassiana* virulence. The length of “the spring stage” was inversely proportional to the virulence of the different *B. bassiana* strains. The longer the “spring stage” was, the weaker the virulence of *B. bassiana* was.

At the same time, on the 5th day after *B. bassiana* CB infection (the day before the peak of population death), *Pseudomonas* (22.54% ± 0.27%), *Cyanobacteria_c_norank* (21.96% ± 0.22%), *Aquabacterium* (12.26% ± 0.70%), *Bacillus* (8.73% ± 0.06%) and *Sphingobium* (6.42% ± 0.40%) were the top five most abundant bacteria in the intestines of house flies.

**Figure 4:**
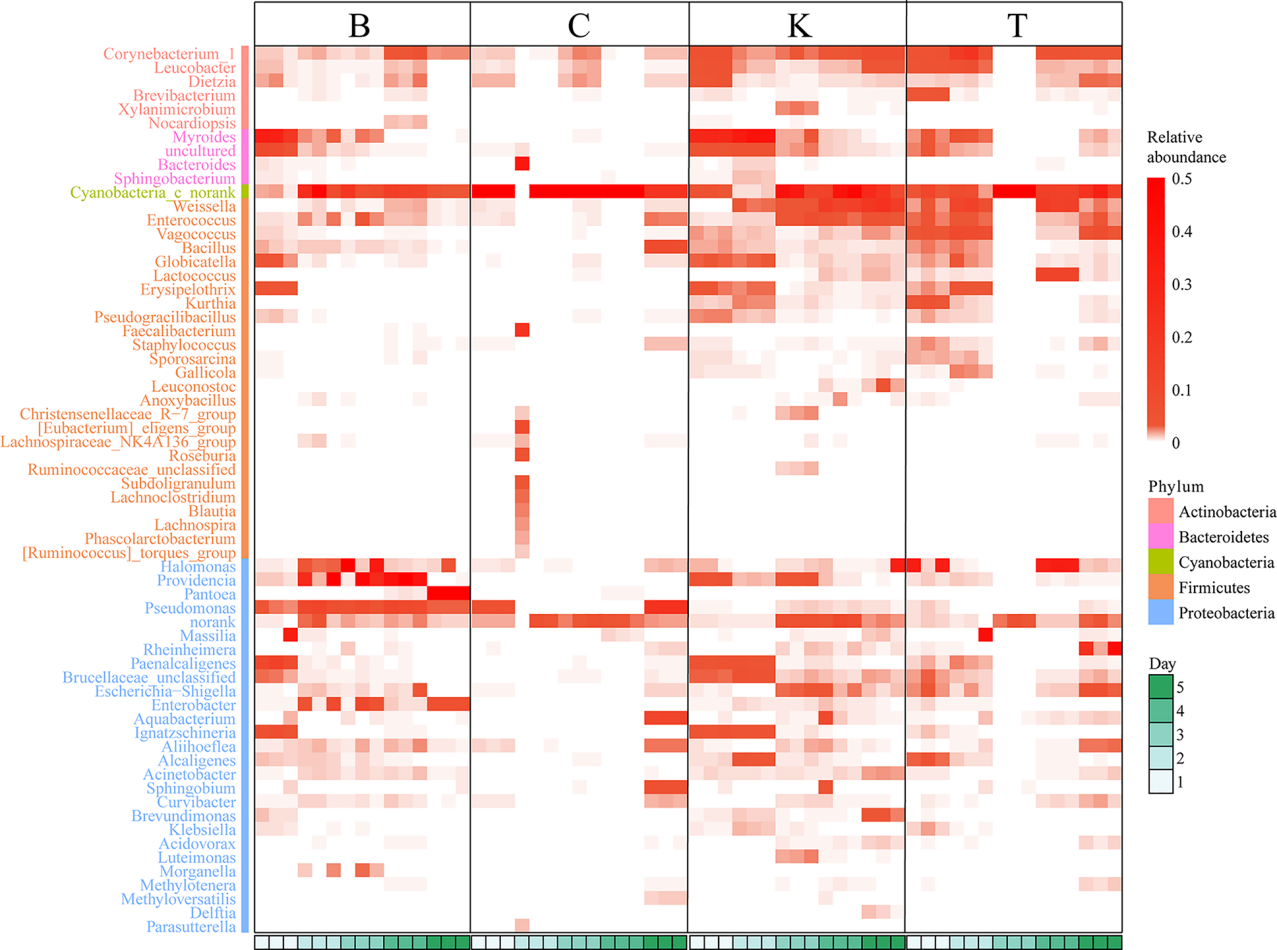
Heatmap based on the intestinal bacterial genera of different house fly groups, color coded by genus following the legend in the panel.

On the 4th day after *B. bassiana* TB infection (the day before the peak of population death), the dominant bacteria in the house fly intestine were *Weissella* (15.98% ± 1.49%), *Cyanobacteria_c_norank* (14.85% ± 0.28%), *Lactococcus* (13.17% ± 0.07%), *Rheinheimera* (27.49% ± 13.78%), *Vagococcus* (5.98% ± 2.01%) and *Escherichia shigella* (4.61%) ± 1.50%) (**Figure 4**, **Figures S1** and **S2**).

No dysbacteriosis was found in the control group house flies (K group); the bacterial group with the largest proportion at 1 day and 2 days was *Myroides*, the proportions were 28.03% ± 0.92% and 37.93% ± 2.50%, respectively. The bacterial group with the largest proportion at 3 and 4 days was *Cyanobacteria_c_norank*, with abundances of 29.80% ± 14.73% and 33.15% ± 17.01%, respectively. The bacterial group with the largest proportion on the fifth day was *Weissella*, representing approximately 19.28% ± 9.19% of the total intestinal bacteria.

Therefore, when *B. bassiana* infects house flies, it will first cause disturbance of the intestinal flora of the house flies, and some kinds of bacteria will multiply to large numbers (such as *Pantoea* in group B or *Cyanobacteria_c_norank* in groups C and T), while disturbance of the intestinal flora generally occurs two days before the population death peak of house flies.

**Figure 5:**
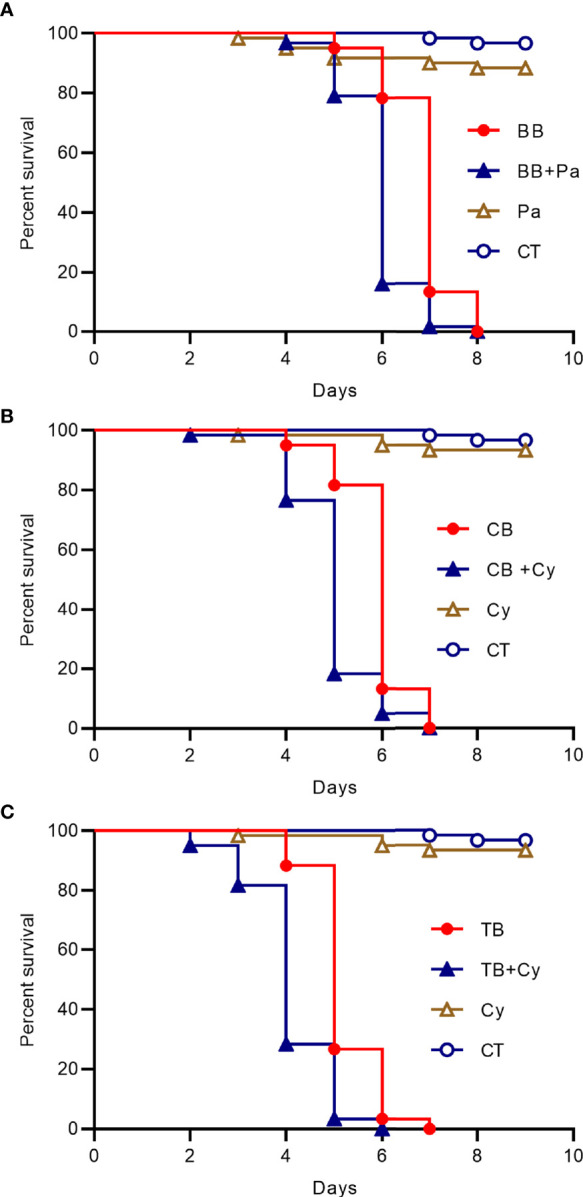
Survival curve of house flies coinfected with B. bassiana and intestinal bacteria. **(A)** *B. bassiana* BB- and Pantoea-coinfected house flies. **(B)** *B. bassiana* CB- and Cyanobacteria-coinfected house flies. **(C)** *B. bassiana* TB- and Cyanobacteria-coinfected house flies.

Then we added the *Cyanobacteria* to the drinking water of the house fly groups with *B. bassiana* CB and TB infection or *Pantoea* was added to the drinking water of the house fly group infected with *B. bassiana* BB, the peak time of death was one day earlier than that of the groups without such addition (**Figure 5**). So the dominant taxa in the intestines of bacteria flora disturbed house flies has a superposition effect with *B. bassiana* infection.

### Estimates of and Variation in Local Microbial Diversity Among Samples From Different Groups of House Flies

There were 268 common genera of bacteria in the 4 groups of house fly intestines. Group B shared 5, 11 and 53 bacterial genera with groups C, T, and K, respectively; group C shared 12 and 19 bacterial genera with groups T and K, respectively; and groups T and K shared 45 bacterial genera. Groups B, C and T shared 5 bacterial genera (**Figure S3**).

**Figure 6:**
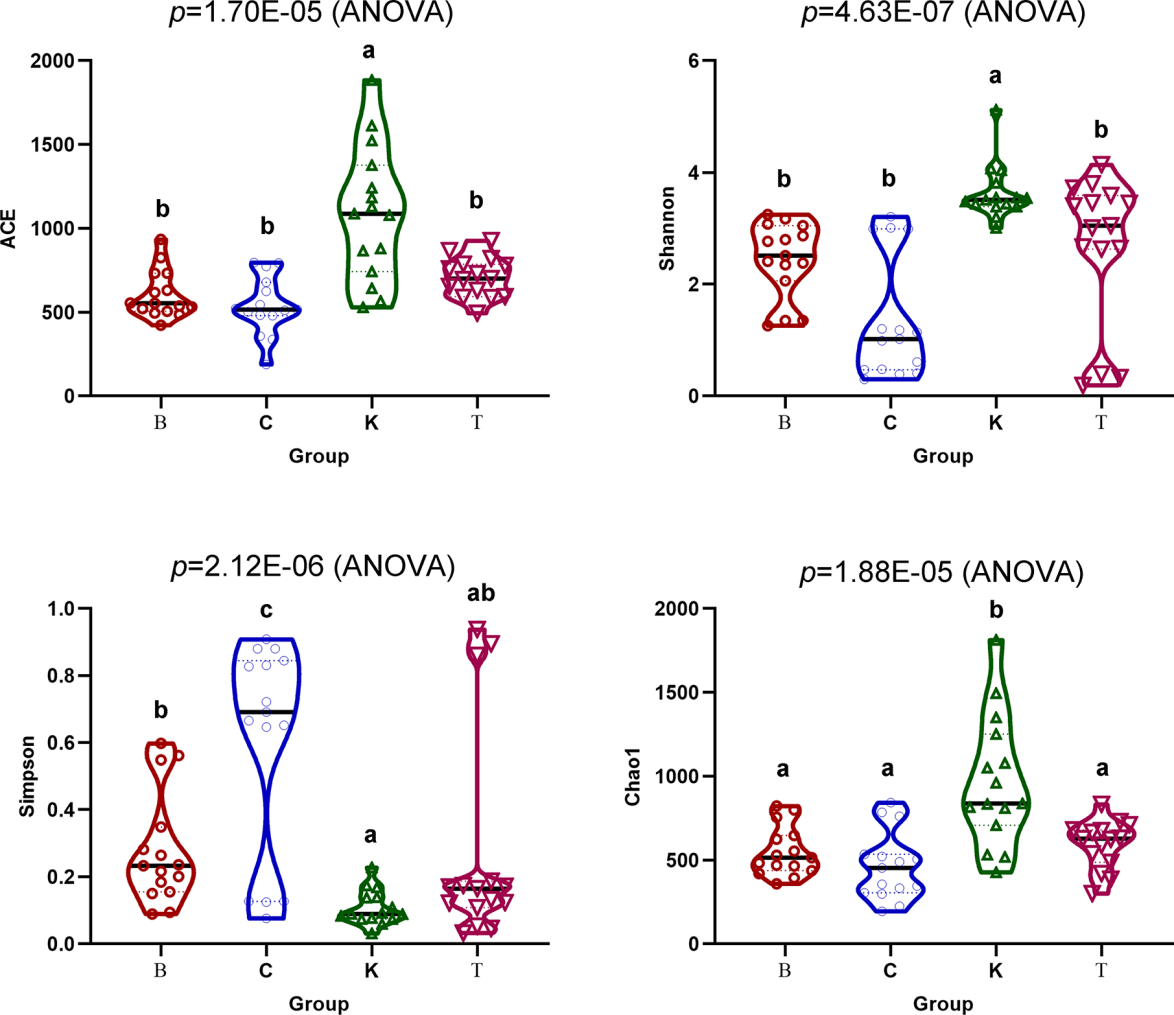
Ace, Chao1, Shannon and Simpson indexes of the intestinal bacteria in the four groups of house flies.

The Chao1 (*p* = 1.70E−05) and Ace (*p* = 4.63E−07) indexes of intestinal bacteria from group K house flies were higher than those from house flies infected by *B. bassiana*, but the two indexes were not significantly different among the three infected groups. Therefore, the bacterial community richness of the house fly intestine decreased after *B. bassiana* infection. The Shannon index (*p* = 2.12E−06) and Simpson index (*p* = 1.88E−05) differed significantly among the four groups. The Shannon index of group K was significantly higher than those of groups B and C but not significantly different from that of group T, and the index was not significantly different among groups B, C and T. The Simpson indexes of groups K, B and T were significantly higher than that of group C (**Figure 6**). However, regarding the dynamics of the Ace index and Chao1 index of the intestinal bacteria in the four groups, the indexes of K2, K3, and K4 were higher than those of the other groups on the same day. Regarding the Shannon index, groups C1 and C2 were significantly lower than the other groups on the same day. The Shannon indexes of groups T3, C4, and B5 were significantly higher than those of the other groups on the same day, but the Simpson indexes of groups T3, C4, and B5 were significantly lower than those of the other groups on the same day (**Figure S4**).

**Figure 7:**
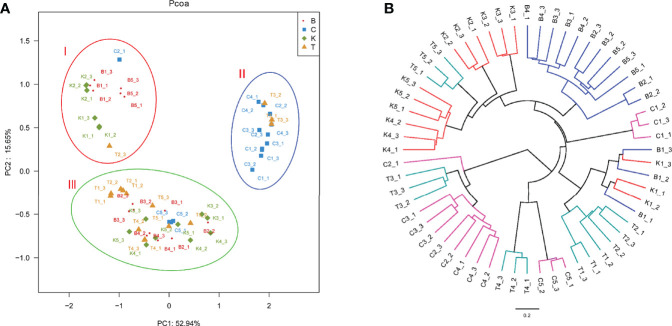
Differences in bacterial community structure and relationships between all of the groups. **(A)** Principal coordinate analysis (PCoA) of bacterial community structure in the four groups. Each symbol represents one sample of intestinal bacteria. **(B)** UPGMA tree analysis of samples during evolution.

The PCoA of bacterial structures in different groups of house fly intestines showed that the samples were divided into three groups: samples B1, B5, K1, and K2 comprised group I, samples C1, C4, C2, and T3 comprised group II, and the remaining samples comprised group III (**Figure 7**). The relationships between the community structures revealed by PCoA were further tested using an unweighted pair-group method with arithmetic mean (UPGMA) tree. The results showed that the samples were mainly divided into two branches: groups B and K and groups C and T were clustered together. However, when subdivided, the clustering analysis result was consistent with the PCoA result (**Figure 7**). Therefore, the bacterial composition of weakly virulent BB strain-infected house flies was similar to that of the control group (group K). The bacterial compositions of the house flies infected with the strongly virulent CB and TB strains were more similar.

### The Network of Bacteria in Different House Fly Intestines

**Figure 8:**
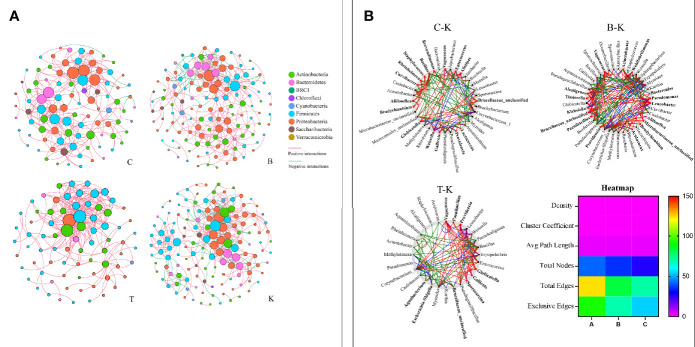
The networks **(A)** and cooccurrence networks **(B)** based on the intestinal microbiomes of intra- and intergroups. **(A)** Intestinal microbiome networks of groups C, B, K and T. **(B)** Potential “driver taxa” of infection based on bacterial network analysis of the infected groups B, C, and T and control group K, marked as B-K, C-K and T-K, respectively. Node sizes are proportional to their scaled NESH (neighbor shift) score (a score identifying important microbial taxa in microbial association networks), and the nodes colored red are important driver taxa. As a result, large red nodes denote particularly important driver taxa in the *B. bassiana* infection process. Line colors indicate node (taxa) connections as follows: association present only in infected groups (red edges), association present only in the control group (green edges), and association present in both infected and control groups (blue edges). The heatmap **(B)** represents the difference in the cooccurrence networks among B-K, C-K and T-K based on the six parameters density, cluster coefficient, average path length, total nodes, total edges and exclusive edges.

The cooccurrence networks of group K were initially much larger than those of groups B, C and T, having larger numbers of nodes and links, a longer average path distance, and larger maximal betweenness (**Figure 8A** and **Table S1**). The percentages of negative interactions between intestinal bacteria from group B, C and T house flies were 15.30%, 4.07% and 0, respectively. However, the percentage for group K house flies was approximately 29.69%. Therefore, the pathogenicity of *B. bassiana* was related to its ability to regulate the intestinal bacterial composition of its hosts.

On the basis of “NetShift” analysis, we speculated that *Vagococcus, Alcaligenes, Bacteroides, Aliihoeflea, Paenibacillus, Tissierella, Microbacteriaceae_*unclassified, and *Leucobacter* were the key bacterial genera associated with the pathogenicity of *B. bassiana* BB; *Gallicola* and *Providencia* were the key bacterial genera associated with the pathogenicity of *B. bassiana* CB; and *Vagococcus, Paenibacillus, Providencia, Gallicola*, and *Sporosarcina* were very important in the pathogenicity of *B. bassiana* TB (**Figure 8B**). Regarding network indexes, the density, cluster coefficient and average path length indexes showed no differences in the comparisons of group B, C, and T cooccurrence networks with group K cooccurrence networks, but the total node, total edge and exclusive edge indexes were different (**Figure 8B**).

## Discussion

Insect mycopathogens produce compounds that inhibit the growth of competing microbes in the host. In many cases, the compounds depend on the developmental stage of the pathogen, including enzymes, elicitors, and secondary metabolites (24). For example, after penetrating the cuticle and peritrophic membranes of host insects, many dimorphic mycopathogens produce yeast-like hyphal bodies that grow exponentially in the nutrient-rich hemolymph (33). Once the hyphal bodies in the hemolymph reach a quorum density, they synchronously convert to the mycelial phenotype (34). Late-stage mycosis in host insects is marked by host death, the digestion of the insect tissue, and concomitant conversion of insect tissue to fungal biomass. During this late growth stage, insect mycopathogens must suppress the resident host microflora, including the often complex microbiome associated with the digestive tract (24, 25).

*B. bassiana* also manipulates the compounds of gut bacteria to produce benefits for its infection; for example, *Serratia marcescens* in the gut of *Anopheles stephensi* is an accomplice of parasitic *B. bassiana*. Once the gut bacteria are removed with antibiotics, the pathogenicity of *B. bassiana* to *Anopheles* declines (25). On the other hand, the gut bacteria of host insects also function as antagonists of *B. bassiana* (5, 27, 35). Therefore, the interactions between *B. bassiana* and host gut bacteria are highly uncertain.

We tested the virulence of three different *B. bassiana* strains to house flies. The virulence of the TB strain was the strongest, followed by those of the CB and BB strains. The population death peaks of house flies infected by the *B. bassiana* strains TB, CB and BB occurred 5, 6, and 7 days after infection, respectively. Until 6 and 7 days after infection by the *B. bassiana* TB and CB strains, all the house flies died of infection, but the cumulative mortality of house flies infected by *B. bassiana* BB was 93.17% until 9 days after infection. Next, we used the three strains of *B. bassiana* to infect house flies and measured the dynamic changes in the intestinal bacteria of infected and control house flies. We found that *B. bassiana* strains with different pathogenicities had different abilities to regulate the intestinal bacterial composition of house flies, and the stronger the virulence of the strain was, the stronger the ability of the strain to regulate the intestinal bacteria of house flies was.

According to high-throughput sequencing, the composition of intestinal bacteria in different infection groups and the control group of house flies was analyzed. Overall, the Ace, Chao1 and Shannon indexes of the intestinal bacteria in *B. bassiana*-infected house flies (groups B, C, and T) were significantly lower, but the Simpson index was higher, than those in the control group (**Figure 6**). However, in terms of the dynamics of the bacterial community indexes, the changes in the indexes were the most obvious from 2 to 4 days after *B. bassiana* infection (**Figure S4**). Cluster analysis and PCoA showed that the composition of the intestinal bacteria of *B. bassiana* BB strain-infected house flies was more similar to that of the control group than to those of the CB- and TB-infected groups, which reflected that different *B. bassiana* strains had different abilities to regulate the intestinal bacteria of their host (**Figure 8**).

We found that the infection of *M. domestica* by *B. bassiana* can cause intestinal bacterial flora disturbance, which mainly manifests as certain bacteria becoming absolutely dominant in the intestines. For *B. bassiana* strain BB-infected house flies, *Pantoea* was the dominant bacterial group when intestinal bacterial flora disturbance occurred, but for *B. bassiana* strain CB- and TB-infected house flies, *Cyanobacteria_c_norank* was the dominant bacterial group causing intestinal bacterial flora disturbance. The two bacteria had synergistic effects on *B. bassiana* infection and the death of house flies (**Figure 5**).

*Pantoea* are known plant pathogens and conditional bacteria of humans (36). However, in insects, *Pantoea* are intestinal commensal bacteria. They are required for the completion of the life cycle in stinkbugs (37), produce compounds that attract insects (38, 39), synthesize key components of the locust aggregation pheromone (40), stimulate spawning (41), and are related to the anti-infection of entomopathogenic fungi (42–46). In house flies, after infection for 5 days by *B. bassiana*, *Pantoea* became the absolutely dominant bacteria in the intestine. Dysbacteriosis of the intestinal flora caused by the proliferation of *Pantoea* may be an important reason for the death of adult house flies caused by *B. bassiana* infection, but the pathogenicity mechanism needs to be further studied.

*Cyanobacteria* are important bacteria that cause water blooms in fresh water. They can produce a series of natural toxins (cyanotoxins) that endanger human health (47). However, in insects, *Cyanobacteria_c_norank* are the predominant bacteria of *Bombyx mori* and *Antheraea pernyi*, although they are probably obtained by feeding on mulberry leaves (48). However, for house flies, the source of *Cyanobacteria_c_norank* is not clear. Regarding the early stage of infection by the TB strain in house flies, *Cyanobacteria_c_norank* was not the main bacterial group, but on the 3^rd^ day after infection, *Cyanobacteria_c_norank* become the most abundant bacterial group of the intestinal flora. Therefore, *Cyanobacteria_c_norank* is not a contaminating bacterial group.

The period from the time of exposure to intestinal flora disturbance was as elastic as a spring; therefore, we named this period the “spring stage”. In the “spring stage, the time from spore germination to invasion of the host is variable between isolates (data not shown), and could be influenced by several virulence factors during cuticle adhesion, penetration, and insect body invasion. The longer the “spring stage” was, the weaker the *B. bassiana* strain virulence was. However, the other time period, from intestinal disturbance of house flies to the population death peak, was fixed to 2 days for the three *B. bassiana* strains. Therefore, the “spring stage” is the key stage in which *B. bassiana* kills house flies, which determines the lethality rates against house fly populations. As reported by Farooq& Freed, the *B. bassiana* strains Bb-01, Bb-08, and Bb-10 had different LT50 values for house flies, perhaps because the three *B. bassiana* strains also had different “spring stages”. The same result was reported in the tick *Haemaphysalis longicornis* (49).

*Staphylococcus* and *Weissella* were the dominant bacterial genera in the intestine of house flies in a previous study (50), but in our results, *Weissella* was relatively consistent throughout the early adult stage of house flies, whereas *Staphylococcus* was not a dominant genus. In the mosquito *Anopheles stephensi*, after infection by *B. bassiana*, the opportunistic pathogenic bacterium *Serratia marcescens* overgrew in the midgut and translocated to the hemocoel, which promoted the death of *Anopheles* (25). However, for *Blattella germanica* and *Delia antiqua*, the gut bacteria enhanced the antagonism of larvae to *B. bassiana* (5, 26, 35). Therefore, the intestinal bacteria of insects are a double-edged sword against infection by *B. bassiana* or other pathogenic fungi. In some insects, they can help resist the infection, while in other insects, they may promote the process of infection.

On the basis of “NetShift” analysis, we speculated that *Vagococcus, Alcaligenes, Bacteroides, Aliihoeflea, Paenibacillus, Tissierella, Microbacteriaceae_*unclassified and *Leucobacter* were the key bacterial genera for the pathogenicity of *B. bassiana* BB; *Gallicola* and *Providencia* were the key bacterial genera for the pathogenicity of *B. bassiana* CB; and *Vagococcus, Paenibacillus, Providencia, Gallicola* and *Sporosarcina* were very important in the pathogenicity of *B. bassiana* TB. Regarding network indexes, the density, cluster coefficient and average path length indexes showed no differences among the cooccurrence networks in the B-K, C-K and T-K comparisons, but the total node, total edge and exclusive edge indexes were different (**Figure 8B**).

**Figure 9:**
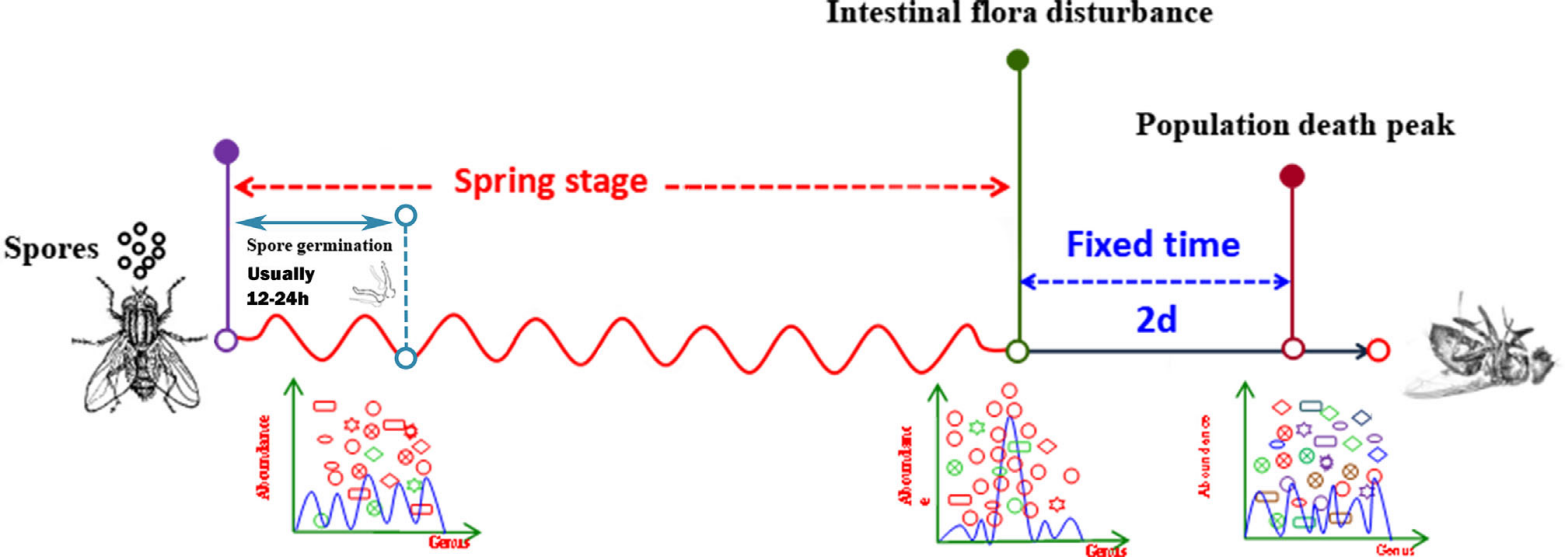
Pattern diagram of the process of *B. bassiana* infecting house flies.

In conclusion, infection by *B. bassiana* altered the composition of the house fly gut bacteria flora. There were three main characteristics. First, the diversity and richness of the intestinal bacteria decreased. Second, a few days after infection, intestinal flora disorder occurred, and the time from infection to dysbacteriosis of the intestinal flora was elastic, similar to a retractable spring. Therefore, we named the elastic period from infection to dysbacteriosis the “spring stage”. The time from the “spring stage” to the death peak of the infected population was fixed to 2 days. Therefore, from infection, the population death peak is elastic, which mainly depends on the length of the “spring stage”. Our research shows that the stronger the virulence of the *B. bassiana* strain is, the shorter the “spring stage” is. Third, the cooccurrence network of the intestinal bacteria of adult house flies was altered after infection by different *B. bassiana* strains, which was mainly manifested as fewer nodes and links and shorter average path distances. However, the positive interaction ratio between the intestinal bacteria increased; the stronger the virulence was, the higher the positive interaction ratio between intestinal bacteria was. Therefore, the disturbance of intestinal flora is an important reason for the death of house fly after infected by *B. bassiana*. The “spring stage” can be used as an important factor for investigating the virulence of *B. bassiana*. The shorter the “spring stage” is, the stronger the virulence is (**Figure 9**).

## Data Availability Statement

The original contributions presented in the study are included in the article/**Supplementary Material**. Further inquiries can be directed to the corresponding authors.

## Author Contributions

RZ and ZZ conceived the project. RZ, ZZ, SF, and XX designed the experiments. SF, XX, ZH, QW, and SW performed the experiments. RZ, SW, and ZZ analyzed the data. RZ and ZZ wrote the manuscript. All authors contributed to the article and approved the submitted version.

## Funding

This work was supported by the National Natural Science Foundation of China (nos. 81572028 and 81871686). The funders had no role in study design, data collection and analysis, decision to publish, or preparation of the manuscript.

## Conflict of Interest

The authors declare that the research was conducted in the absence of any commercial or financial relationships that could be construed as a potential conflict of interest.
