# Supplementary material for: Dysbacteriosis of the Intestinal Flora Is an Important Reason for the Death of Adult House Flies Caused by Beauveria bassiana
Source: Front Immunol. 2021 Jan 26;11:589338. doi: 10.3389/fimmu.2020.589338 (PMC7871782; doi:10.3389/fimmu.2020.589338)

Fig. S1 Relative abundances of the top 8 phyla (A) and top 43 families (B) of intestinal bacteria in houseflies infected by the three strains of *Beauveria bassiana* and control houseflies.

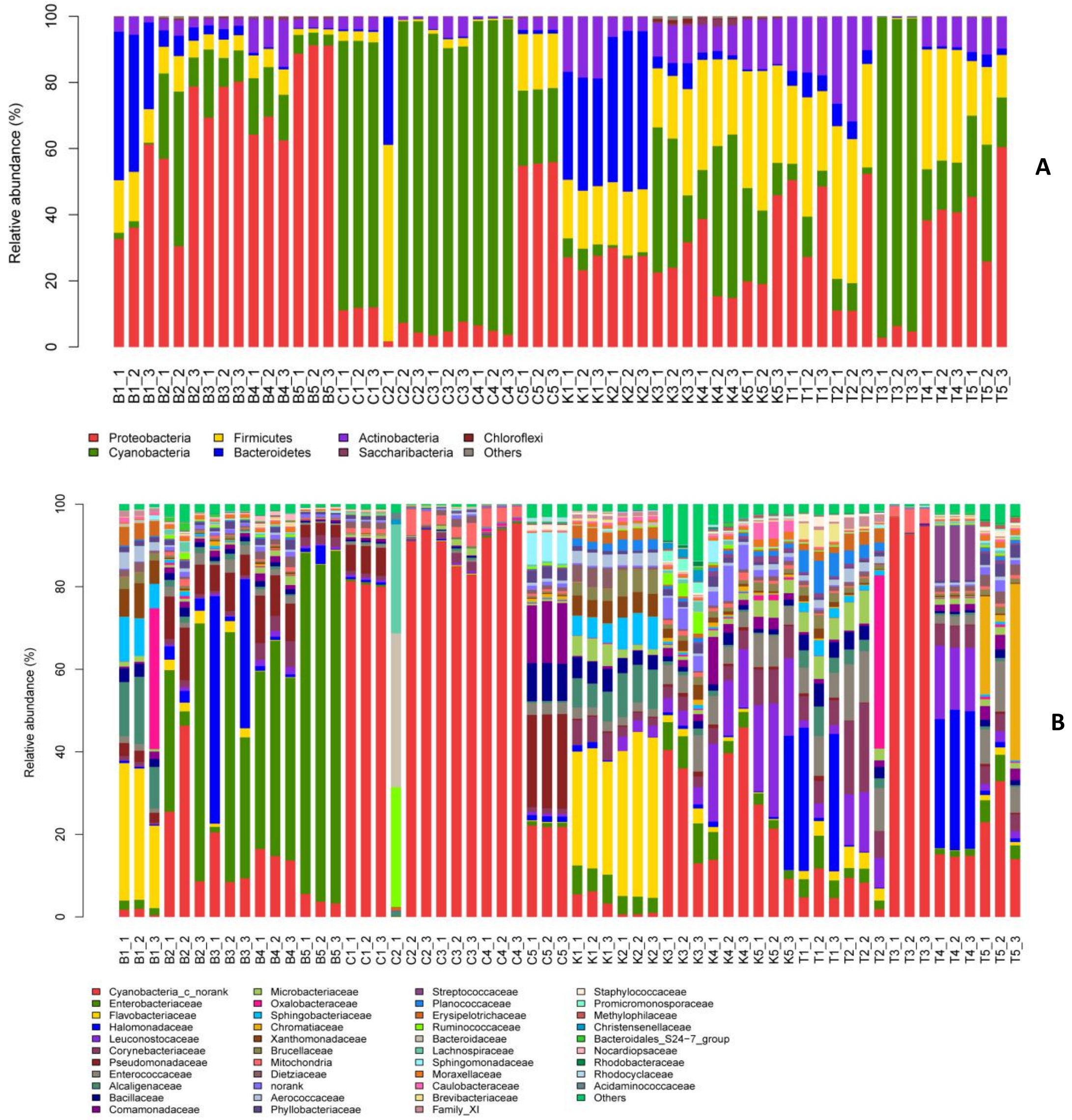

Supplement: Supplementary file 1 [file DataSheet_1.pdf]
