# Supplementary figures and images for: Dysbacteriosis of the Intestinal Flora Is an Important Reason for the Death of Adult House Flies Caused by Beauveria bassiana
Source: Front Immunol. 2021 Jan 26;11:589338. doi: 10.3389/fimmu.2020.589338 (PMC7871782; doi:10.3389/fimmu.2020.589338)

**Fig. S2 Dynamics of key bacterial OTU numbers in different groups.**

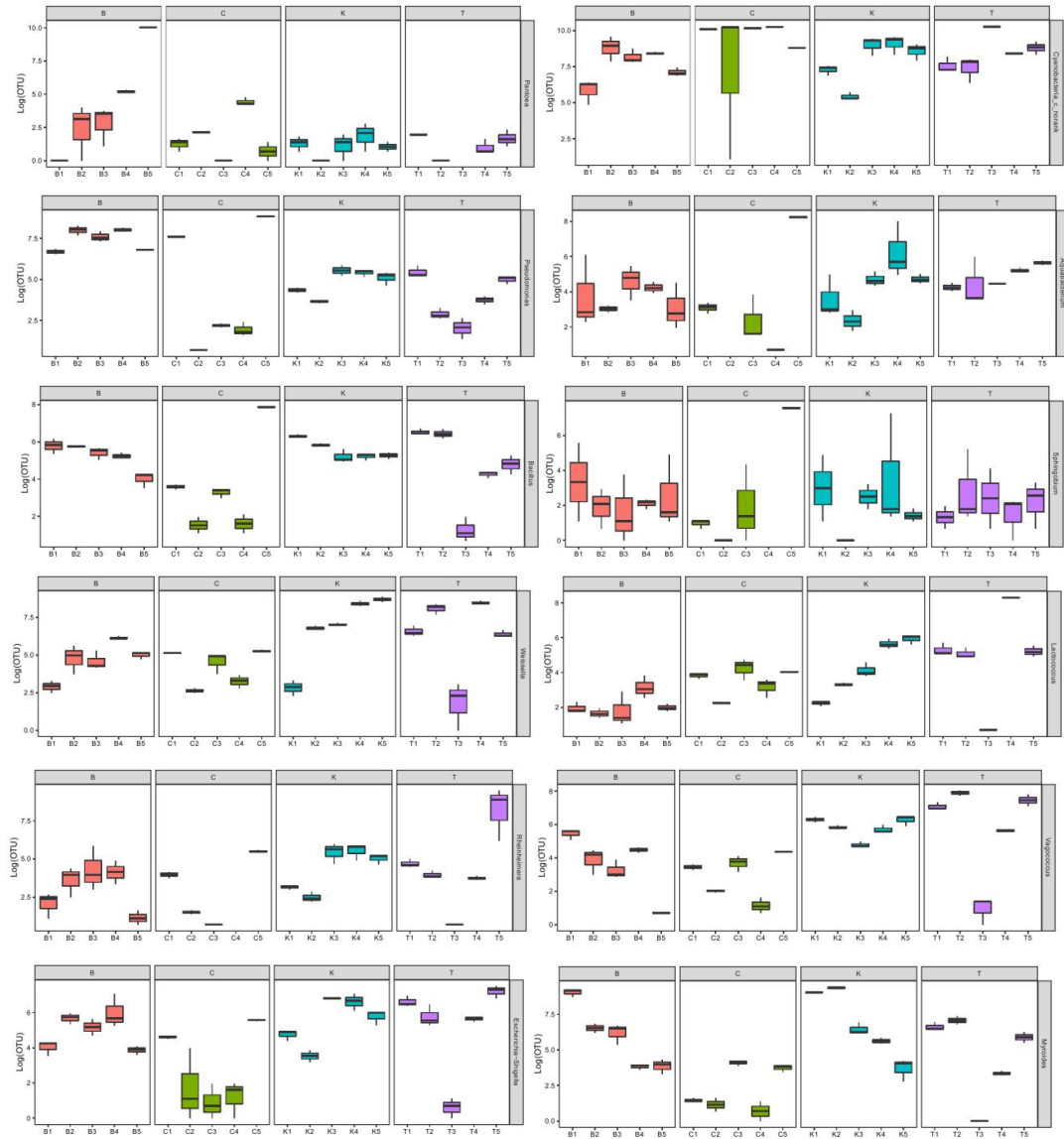

Supplement: Supplementary file 2 [file DataSheet_2.pdf]

Fig. S3 The Venn diagram of intestinal bacteria of houseflies in each group.

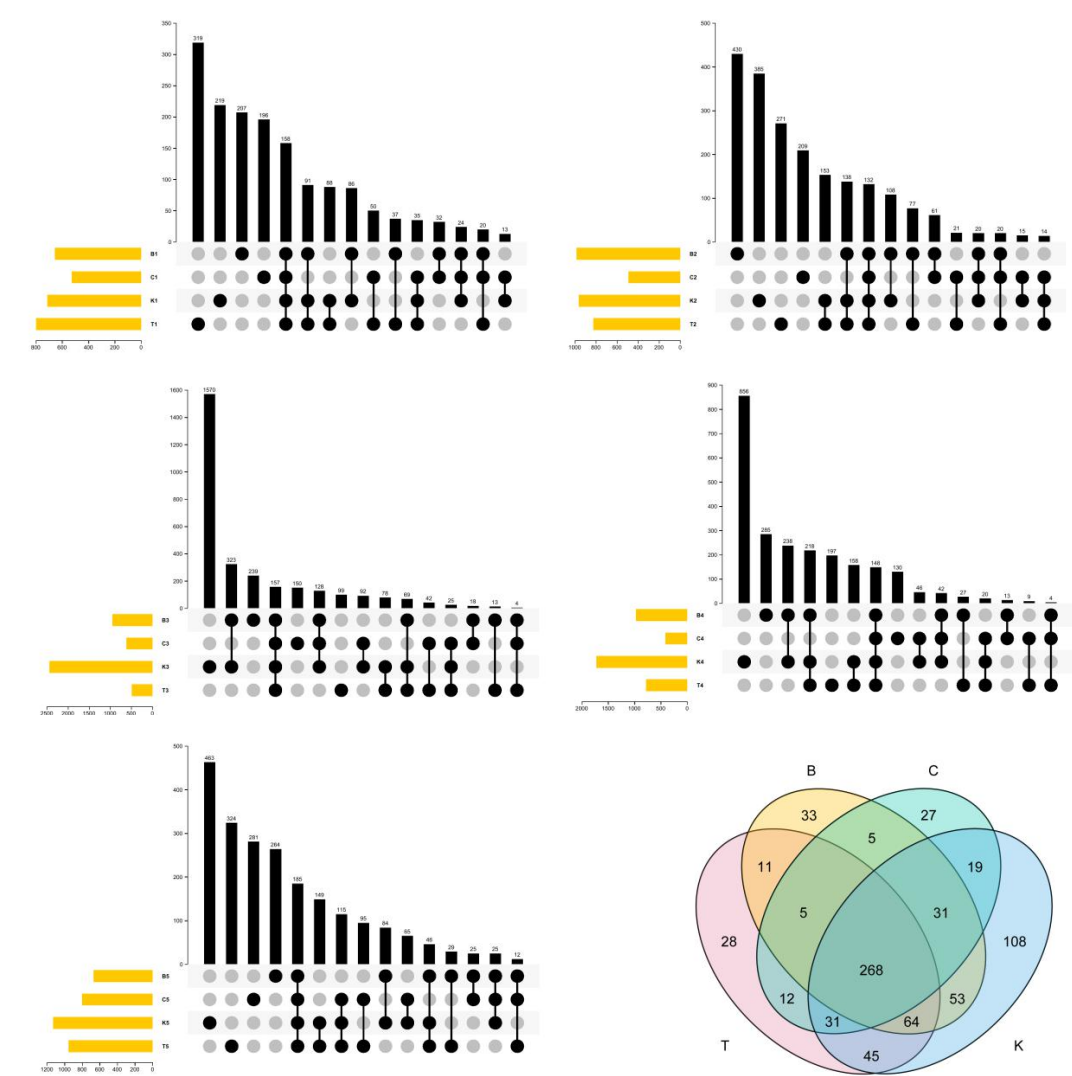

Supplement: Supplementary file 3 [file DataSheet_3.pdf]

**Fig. S5 Collection sites (A) and phylogenetic tree (B) of the three *B. bassiana* strains BB, CB and TB.**

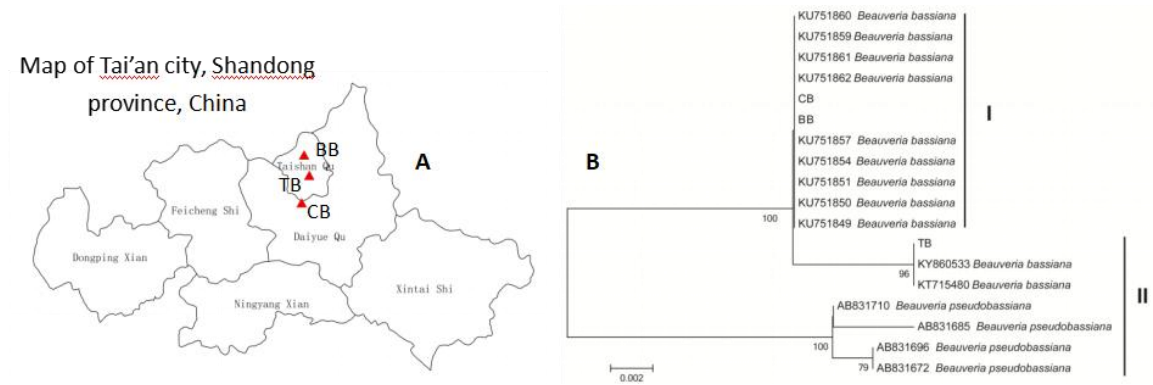

Supplement: Supplementary file 5 [file DataSheet_5.pdf]
