# Supplementary material for: Dysbacteriosis of the Intestinal Flora Is an Important Reason for the Death of Adult House Flies Caused by Beauveria bassiana
Source: Front Immunol. 2021 Jan 26;11:589338. doi: 10.3389/fimmu.2020.589338 (PMC7871782; doi:10.3389/fimmu.2020.589338)

Fig. S4 Dynamics of the Ace, Chao1, Shannon and Simpson indexes of intestinal bacteria in the four groups of houseflies.

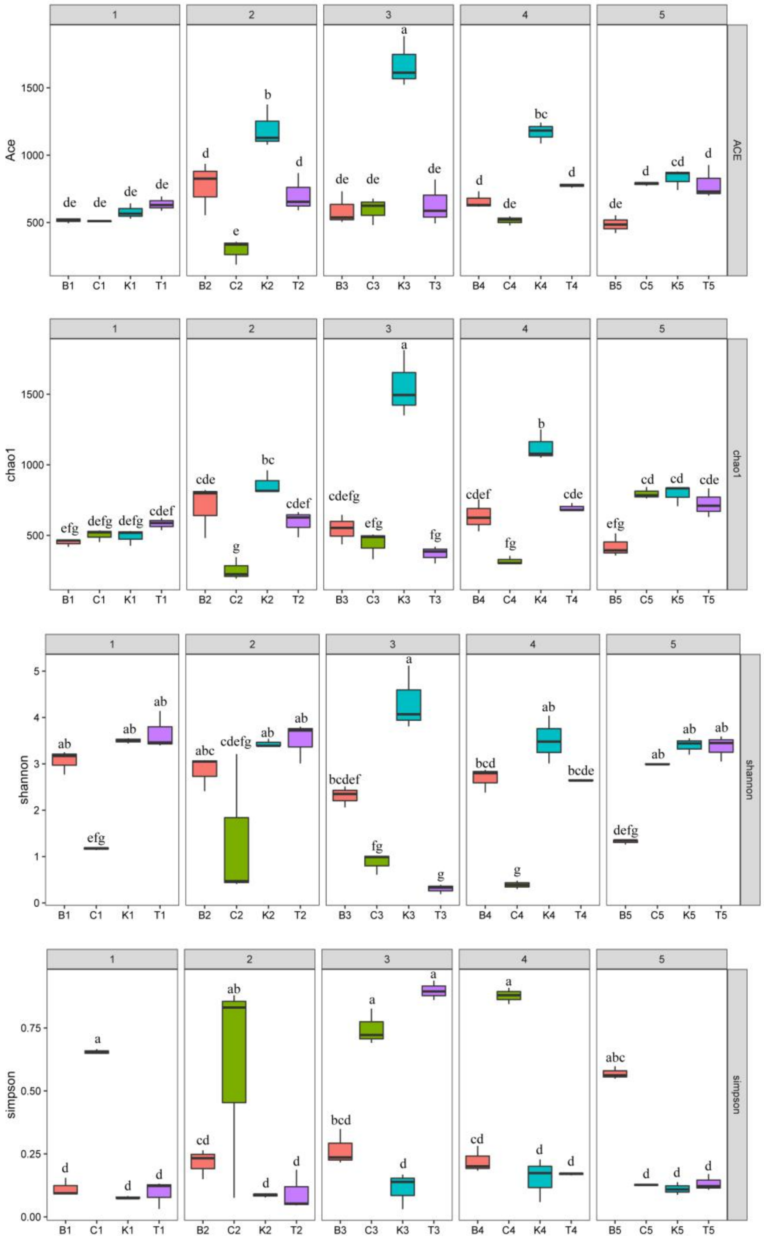

Supplement: Supplementary file 4 [file DataSheet_4.pdf]
