# Supplementary material for: Dysbacteriosis of the Intestinal Flora Is an Important Reason for the Death of Adult House Flies Caused by Beauveria bassiana
Source: Front Immunol. 2021 Jan 26;11:589338. doi: 10.3389/fimmu.2020.589338 (PMC7871782; doi:10.3389/fimmu.2020.589338)
Supplement: Supplementary file 6 [file DataSheet_6.pdf]

Table S1 The cooccurrence networks indexes of different groups.

Index	Group			
	B	C	K	T
Network index	0.790	0.860	0.890	0.950
Total nodes	130	93	143	77
Total links	353	269	384	240
R square of power-law	0.83	0.723	0.84	0.751
Average degree (avgK)	5.431	5.785	5.371	6.234
Average clustering coefficient (avgCC)	0.292	0.322	0.298	0.225
Average path distance (GD)	3.705	3.276	4.02	3.718
Geodesic efficiency (E)	0.333	0.369	0.313	0.378
Harmonic geodesic distance (HD)	3	2.708	3.199	2.645
Maximal degree	25	19	23	29
Nodes with max degree	OTU17	OTU70	OTU31	OTU2
Centralization of degree (CD)	0.154	0.147	0.126	0.308
Maximal betweenness	1278.743	704.966	2871.965	925.733
Nodes with max betweenness	OTU21	OTU21	OTU5	OTU43
Centralization of betweenness (CB)	0.139	0.147	0.271	0.299
Maximal stress centrality	8933	4375	26184	28619
Nodes with max stress centrality	OTU27	OTU199	OTU5	OTU43
Centralization of stress centrality (CS)	0.966	0.929	2.478	9.318
Maximal eigenvector centrality	0.342	0.31	0.27	0.329
Nodes with max eigenvector centrality	OTU17	OTU70	OTU23	OTU21
Centralization of eigenvector centrality (CE)	0.3	0.24	0.227	0.259
Density (D)	0.042	0.063	0.038	0.082
Reciprocity	1	1	1	1
Transitivity (Trans)	0.377	0.319	0.377	0.268
Connectedness (Con)	0.796	0.916	0.827	0.827
Efficiency	0.956	0.942	0.962	0.915
Hierarchy	0	0	0	0
Lubness	1	1	1	1
